# A Comparative Study of HOTAIR Expression in Breast Cancer Patient Tissues and Cell Lines

**DOI:** 10.22074/cellj.2020.6543

**Published:** 2019-10-14

**Authors:** Asghar Arshi, Farzaneh Raeisi, Esmaeil Mahmoudi, Fatemeh Mohajerani, Hamidreza Kabiri, Razieh Fazel, Maedeh Zabihian-Langeroudi, Amela Jusic

**Affiliations:** 1. Young Researchers and Elite Club, Najafabad Branch, Islamic Azad University, Najafabad, Iran; 2. Young Researchers and Elite Club, Shahrekord Branch, Islamic Azad University, Shahrekord, Iran; 3. Department of Medical Physics, School of Medicine, Isfahan University of Medical Sciences, Isfahan, Iran; 4. Department of Genetics, Faculty of Modern Medical Science, Islamic Azad University of Medical Sciences of Tehran, Tehran, Iran; 5. Student Research Committee, Jahrom University of Medical Sciences, Jahrom, Iran; 6. Department of Biology, Tonekabon Branch, Islamic Azad University, Tonekabon, Iran; 7. Department of Biology, Faculty of Natural Sciences and Mathematics, University of Tuzla, Tuzla, Bosnia and Herzegovina

**Keywords:** Breast Cancer, Cell Line, *HOTAIR* lncRNA, Quantitative Reverse-Transcription Polymerase Chain Reaction

## Abstract

**Objective:**

Recent data suggest that increased levels of the *HOTAIR* long non-coding RNA (lncRNA) are involved in the development of various types of malignancy, including breast cancer. The aim of present study was to investigate *HOTAIR* lncRNA expression profile in breast cancer (BC) patients and cell lines.

**Materials and Methods:**

In this experimental study, expression level of *HOTAIR* lncRNA was evaluated in BC and normal tissues of 15 patients as well as MDA-MB-231, MCF-7 and MCF-10A cell lines, using quantitative reverse- transcription polymerase chain reaction (qRT-PCR). *HOTAIR* lncRNA expression levels were estimated using 2^-ΔΔCt^ method. Further, receiver operating characteristic (ROC) curve analysis was done to evaluate the selected lncRNA diagnostic potential. The Cox’s proportional hazards regression model was performed to evaluate the predictive value of this lncRNA level in BC patients.

**Results:**

The results of present study demonstrated no significant difference in the expression of *HOTAIR* lncRNA in MCF7 and MDA-MB-231 cancer cell lines compared to MCF-10A as normal cell line (P>0.05). However, we observed a significantly increase in the expression of *HOTAIR* in BC patients compared to normal tissues (P<0.001). Significant associations were found between gene expression and tumour size and margin. We found 91.1% sensitivity and 95.7% specificity of circulating *HOTAIR* with an area under the ROC curve of 0.969. The Kaplan-Meier analysis indicated significant correlation between *HOTAIR* expression and overall survival.

**Conclusion:**

This study demonstrated that expression of *HOTAIR* is increased in BC and might be associated with its progression. According to these findings, *HOTAIR* expression could be proposed as biomarkers for BC early diagnosis and prognosis.

## Introduction

Long non-coding RNAs (lncRNAs) are a diverse and large class of non-coding RNA molecules with more than 200 nucleotides length which are mostly transcribed by RNA polymerase II (RNA pol II) (1). lncRNAs play essential role in regulation of different cellular processes. Recently, lncRNAs are reported as key regulators of gene expression and they can act as oncogenes or tumour suppressor genes. According to their oncogenic potential they may play a critical role in oncogenesis, migration, cell differentiation, angiogenesis, apoptosis and proliferation (2). Furthermore, lncRNAs associate with different cancers and malignant behaviour of cancer cells (3). One of the first described lncRNAs is the Hox (homeobox) transcript antisense intergenic lncRNA (*HOTAIR* lncRNA) and they play important role in development of breast cancer (BC) (4). Current studies suggest that *HOTAIR* lncRNA reprograms chromatin state which can promote cancer metastasis (5). *HOTAIR* coordinates in chromatin modification and through which it affects expression of multiple genes involved in various cellular functions (6). Recent studies indicated an association between circadian rhythm (CR) disruption and increased risk of BC development (7). Interestingly, CR disruption has been associated with decreased telomere length, where short telomere length, itself, is correlated with BC development (8).

*HOTAIR* expression levels are significantly high in breast tumours, and its measurement is a determinative indicator of primary breast tumours, possibility of metastasis and patient survival (9). The most commonly used cell line in BC research is MDA-MB-231 which provides essential tools for complex biological expression analysis (10). This cell line is originated from a pleural effusion with a metastatic mammary adenocarcinoma and it is a highly aggressive, used as a model of triple negative BC (TNBC) (11, 12). In the present study, we measured *HOTAIR* lncRNA expression level in BC and normal epithelial tissues, in addition to MCF7 and MDAMB- 231 BC cell lines.

## Materials and Methods

### Breast cancer and normal tissue sample collections

In this experimental study, BC and normal breast tissues from 15 patients (morphologically confirmed by a pathologist) were collected consecutively between July and November 2015 at Shiraz General Hospital (Shiraz, Iran). These 15 patients were included in the present study, according to the inclusion and exclusion criteria. The inclusion criteria were as follows: i. All patients hospitalized during 2015, those with diagnosis or procedure codes related to breast cancer, and ii. Adult patients (19 years of age or older) with cancer of the breast. The exclusion criteria include: i. History of any other malignancy, ii. History of previous relevant treatment, including chemotherapy, radiotherapy or endocrinotherapy, and iii. People with 18 years of age or younger. In this research, ethical considerations were approved based on the International Campus of Shahrekord University of Medical Sciences, Shahrekord, Iran.

### Culture of the cell lines

MDA-MB-231 (ATCC^®^ HTB-26™), MCF-7 (ATCC^®^ HTB22™) and MCF-10A (ATCC^®^ CRL-10317™) cell lines were cultured and maintained in RPMI-1640 medium (Sigma-Aldrich, USA) supplemented with L-Glutamine (Sigma-Aldrich, USA), 10% fetal bovine serum (FBS, Gibco, USA), 100 U/ml penicillin (Sigma-Aldrich, USA) and 100 μg/ml streptomycin (Sigma-Aldrich, USA) in the cell culture incubator at 37˚C, 5% CO_2_ and 95% humidity.

### RNA extraction and cDNA synthesis

Total RNA was extracted from the tissue samples and cell lines, using the RNXTM-Plus solution (SinaClon, Iran) according to the manufacturer’s instructions except the additional step of extended treatment with DNaseI for one hour. Purity, concentration and quality of the extracted RNAs were analysed by Thermo Scientific NanoDrop™ 1000 Spectrophotometer (USA) and electrophoresis on 2% agarose gel. For complementary DNA (cDNA) synthesis, 1 μg RNA was added to PrimeScriptTM-RT reagent kit (TaKaRa, Japan) containing random hexamer priming mix. Concentration of the synthesized cDNA was measured by spectrophotometry.

### Quantitative reverse transcription polymerase chain reaction

*HOTAIR* expression level in BC and normal breast epithelial tissues as well as MDA-MB-231, MCF7 and MCF-10A cell lines was evaluated by quantitative reverse transcription PCR (qRT-PCR) method, using a rotor gene 6000 Corbett detection system (Qiagen, Germany) and SYBR^®^Premix Ex Taq^TM^ II kit (TaKaRa, Japan), according to the manufacturer’s instructions. Thermal cycling amplification was set up according to next protocol: initial activation at 95˚C for 5 minutes followed by 40 cycles at 95˚C for 15 seconds and 65˚C for 1 minute. As control sample nuclease free water was used without adding any template. Melting curve analysis was performed to verify specificity of PCR products. The size and specificity of PCR products were verified by electrophoresis on 2% agarose gel. For qRT-PCR analysis, all samples were normalized to Pumilio RNA Binding Family Member 1 gene (*PUM1*). The qRT-PCR assays were performed in triplicate and the data were presented as the mean ± standard error of mean (SEM). The mean value in each triplicate was used to calculate the relative lncRNA level (ΔCt=Ct mean lncRNAs-Ct mean *PUM1*). Expression fold changes were calculated using 2^-ΔΔCt^ methods.

### Statistical analysis

The relationship between expression of *HOTAIR* and clinical pathological parameters was determined using the chi-square test and Fisher’s exact test. Receiver operating characteristic (ROC) curves were used to assess diagnostic value of the marker. Area under the curve (AUC) was computed for ROC curve. Overall survivals (OS) were presented by the Kaplan-Meier curves, and the log-rank test was used to determine significance between gene expression levels and patient outcome. Data are shown as the means ± SEM and case (%) or number (%). A P value of 5% (*; P<0.05) was considered significant. Statistical analyses were performed by the Graph Pad Prism version 7.00 (Graph Pad Software, USA).

## Results

**Fig 1:**
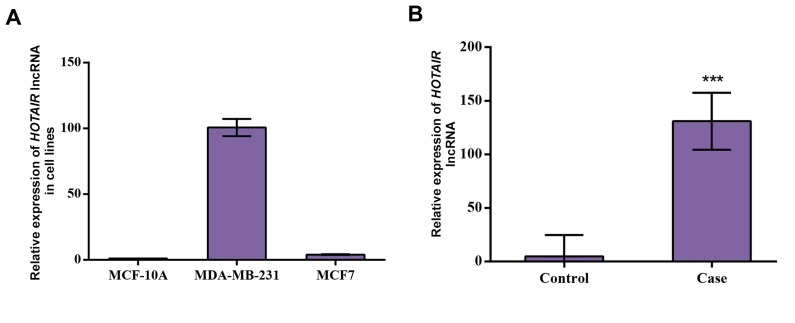
Relative expression of HOTAIR in breast cancer cell lines, case and control samples. A. MDA-MB-231 and MCF-7 cancer cell lines compared to that in MCF-10A control cells. There is no signiﬁcant difference in the expression of HOTAIR in both of MCF-7 and MDA-MB-231 cancer cell lines compared to normal cell line MCF-10A (P>0.05) and B. Breast cancer compared to normal breast tissues. There is a signiﬁcant increase in the expression level of HOTAIR in patients with breast cancer compared to the controls (P<0.001). ***; Significant at the 0.0001 level.

Table 1 summarizes the available patients’ demographic and clinical data. All tissue samples at the time of resection were transferred into RNA-later solution (Sigma-Aldrich, USA) and stored at -20˚C for the further RNA extraction. Applicable international, national and institutional guidelines for the care of human were followed. Figure 1A shows relative expression of *HOTAIR* in MDAMB- 231 and MCF-7 cancer cell lines compared to MCF- 10A control cell line. There is no signiﬁcant difference of *HOTAIR* expression in both of MCF-7 and MDAMB- 231 cancer cell lines compared to normal cell line MCF-10A (P>0.05). The expression level of *HOTAIR* was significantly increased in BC tissue of patients compared to normal tissues (P<0.001, Fig .1B). Correlation between *HOTAIR* expression and the clinical pathological variables of BC cases are shown in Table 2. Signiﬁcant associations were found between gene expression and tumour size and margin.

**Table 1:** Characteristics of the cancer specimens used in this study


Variables	Frequency	Valid percent	Cumulative percent

Age (Y)			
≤47	15	65.2	65.2
˃47	8	34.8	100
Histologic grade			
Well differentiated	1	6.7	6.7
Moderate differentiated	10	66.7	73.3
Poor differentiated	4	26.7	100
Tumour side			
Left	7	46.7	46.7
Right	8	53.3	100
Prevascular invasion			
Negative	4	26.7	26.7
Positive	10	66.7	93.3
Other	1	6.7	100
Preneural invasion			
Negative	3	20	20
Positive	12	80	100
Lymph-node involvement status			
Free	6	40	40
Involved	9	60	100
Total	15	100	
Staging (TNM, Clinical)			
I	8	53.3	53.3
II	7	46.7	100
Total	15	100	


TNM; Tumour, nodes and metastases.

**Table 2:** Correlation of *HOTAIR* expression level and clinical pathological variables in the breast cancer cases


Variables	Cases (%)	HOTAIR lncRNA			P value
		Low	High		

Age (Y), 46.80 ± 2.57 (32-65) (mean ± SE)				0.782
≤47	53.3	26.7		26.7	
>47	46.7	26.7		20	
Tumour grade				0.626
Ι	6.7	6.7		0	
II	66.7	33.3		33.3	
III	26.7	13.3		13.3	
Nuclear grade				0.394
Low	7.1	7.1		0	
High and intermediate	28.6	21.4		7.1	
High	64.3	28.6		35.7	
Tumour stage				0.232
T1	40	26.7		33.33	
T2	13.3	0		13.3	
T3	40	26.7		13.33	
T4	6.7	0		6.7	
Tumour size (cm)				0.029
<2	73.3	26.7		46.7	
≥2	26.7	26.7		0	
Area of invasive component, 4.09 ± 0.13 (0.7-9.5 cm^2^) (mean ± SE)				0.464
<4	66.7	40		26.7	
≥4	33.3	13.3		20	
Tumour side				0.447
Right	53.3	33.3		20	
Left	46.7	20		26.7	
Margin				0.029
Free	73.3	26.7		46.7	
Involved	26.7	26.7		0	
Prevascular Invasion				0.512
Negative	26.7	13.3		13.3	
Positive	73.3	40		33.3	
Preneural Invasion				0.506
Negative	20	13.3		6.7	
Positive	80	40		40	


Bold values indicate P<0.05.

## Diagnostic value of *HOTAIR* in breast cancer

**Fig 2:**
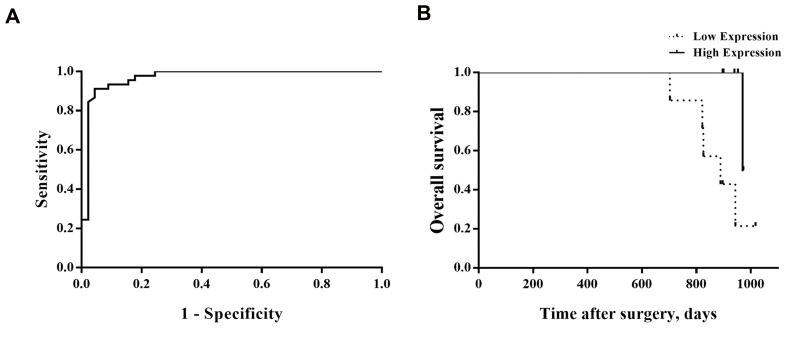
Receiver operating characteristic (ROC) and Kaplan-Meier curves for *HOTAIR* expression. **A.** ROC curve analysis to determine cut-off point for high expression of *HOTAIR*. The area under curve (AUC) was 0.969 (95% CI, 0.933- 1.005) and **B.** Kaplan-Meier curve for BC patients in high-expression (46.7%) and low-expression (53.3%) groups segregated by the cut-off point. BC patients with a low expression level of *HOTAIR* had a poor prognosis (log-rank test, P=0.048).

The ROC curve was created and the AUC was computed to determine capability of the *HOTAIR* expression and difference between cancer and control tissues by calculating sensitivity and specificity for possible cut-off point of *HOTAIR*. The ROC analysis distinguished the optimal cut-off value for *HOTAIR*. We found 91.1% sensitivity and 95.7% specificity for circulating *HOTAIR* with an area under the ROC curve of 0.969 (Fig .2A).

## Correlation of HOTAIR expression with patient survival

In order to assess prognostic value of *HOTAIR* as a BC biomarker, we investigated association of the *HOTAIR* expression levels with survival through Kaplan-Meier analysis. We used the log-rank test in BC patients. The Cox proportional hazards regression model was also used to evaluate predictive value of *HOTAIR* in BC patients. OS was defined as the time between date of surgery and date of death or last follow-up. Clinical pathological factors and OS were then analysed in the high and low level of *HOTAIR* expression groups. Results indicated that low levels of *HOTAIR* have shorter survival time (P>0.048, Table 3, Fig .2B).

**Table 3:** Log rank test for all patients with breast cancer


Variables	Overall survival		P value
	HR	95% CI	

HOTAIR (low vs. high)	6.423	1.128-32.22	0.048
Age (Y), (<47 vs. ≥47)	1.749	0.3501-8.657	0.506
Tumour grade (Ι-II vs. III)	0.560	0.07296-3.501	0.493
Nuclear grade (Low vs. high and intermediate-high)	1.977	0.1596-40.67	0.517
Tumour stage (T1-T2 vs. T3-T4)	0.5745	0.1041-2.891	0.800
Tumour size (<2 cm vs. ≥2 cm)	0.460	0.04656-2.841	0.347
Area of invasive component (<4 cm^2^ vs. ≥4 cm^2^)	0.576	0.07625-3.827	0.378
Tumour side (right vs. left)	0.585	0.1185-2.937	0.525
Margin (free vs. involved)	0.507	0.05886-3.166	0.416
Prevascular invasion (negative vs. positive)	1.605	0.3264-8.170	0.566
Preneural invasion (negative vs. positive)	0.336	0.01890-1.809	0.166


HR; Hazard ratio. Bold values indicate P<0.05.

## Discussion

A number of different genetic and environmental factors that can increase the likelihood of BC have been identified. Accumulated data suggests that lncRNAs play crucial roles in RNA processing, genomic reprogramming, apoptosis, cell proliferation, cell cycle and chromatin modification (13). *HOTAIR* is recognised as a risk factor of various types of tumourigenesis including BC (14). Overexpression of *HOTAIR* may influence tumour formation and induce invasion, migration and proliferation of BC. According to that, altered expression of *HOTAIR* may induce cell proliferation of BC. In this study we evaluated expression level of *HOTAIR* in BC patients and cell lines. Our result showed that *HOTAIR* was up-regulated in BC tissues, while it was not increased in MCF-7 and MDA-MB-231 cell lines.

Regarding the lncRNA functions, BC is one of the most studied malignancies. In has been demonstrated that *HOTAIR* might have a vital role in this regulation due to the interaction with a wide spectrum of miRNAs (15, 16). Recent studies indicated a significant association of *HOTAIR* overexpression with tumour size, advances and extensive metastasis in BC (16-19). *HOTAIR* might act as a gene expression regulator in the BC related to mutations of *BRCA1. HOTAIR* promoter contains several estrogen receptors (ERs), and it has been shown that estradiol regulated *HOTAIR* expression in ER positive BC cells. However, this regulation was abolished in BC cells with inactive ERs, indicating the critical role of these receptors in estradiol-mediated control of *HOTAIR* expression (20). *HOTAIR* and some other lncRNA expression analyses in 164 ER-positive primary BC cases demonstrated that these lncRNAs could be independent prognostic markers (21). Gökmen-Polar et al. (22) indicated that the utility of *HOTAIR* as a prognostic marker in BC is limited to ER-negative cases. Therefore, significant up-regulation of *HOTAIR* obtained from BC tumour samples compared to BC cell lines, in this study, may explain the molecular mechanism causing poor prognosis of this cancer. We found that level of *HOTAIR* expression was not associated with clinical characteristics of BC, while enhanced expression level of *HOTAIR* might associate with tumour size, margins and lower disease relapse. In a research, Lu et al. studied *HOTAIR* expression and methylation of its downstream intergenic CpG islands in 348 samples of primary BC (23). Their results indicated that increased methylation could associate with a worse prognosis in the patient.

According to the results of present study, patients with low level of *HOTAIR* showed shorter survival time. Bhan et al. (20) demonstrated that HOTAIR is critical for survival and proliferation of MCF-7 BC cells. Pádua Alves et al. (24) showed that *HOTAIR* in a BC cell line was a critical regulator of genes involved in epithelial to mesenchymal transition. Up-regulation of HOTAIR has been described as a useful predictor of survival and progression in several cancer types including pancreatic cancer (25), hepatocellular carcinoma (26) oesophageal cancer (27), gastrointestinal stromal (28), nasopharyngeal carcinoma (29) and colorectal cancer (30).

One major obstruction of this study was limited number of BC and control samples enrolled from the same hospital which might not fully substantiate accuracy of the results.

## Conclusion

Taking into consideration that the major problem in early diagnostic and treatment of different types of cancer, including BC, is lack of highly sensitive and specific tumour biomarkers, altered expression levels of *HOTAIR* lncRNA can be a guide for investigation of cancer biomarkers. Thus, our results indicated that *HOTAIR* expression profiling and elucidating its function may be proposed as a useful biomarker for BC diagnosis as well as a therapeutic target in cancer gene therapy. However, further investigations and follow up studies on larger patient samples are required.
